# Circulating Tumor Cells: Emerging Frontiers in Cancer Technology

**DOI:** 10.1017/erm.2026.10039

**Published:** 2026-03-02

**Authors:** Parth Agarwal, Rachana Raman, Manasa Bhagwat, Prasoon Agarwal, Praveen Kumar

**Affiliations:** 1 Manipal Institute of Technology, Manipal Academy of Higher Education, Manipal, India; 2 National Bioinformatics Infrastructure Sweden (NBIS), SciLifeLab, Division of Occupational and Environmental Medicine, Department of Laboratory Medicine, Lund University, Lund, Sweden

**Keywords:** biomarkers, circulating tumor cells, immune surveillance, magnetic immunocapture, nano targeting, stemness

## Abstract

**Background:**

Circulating tumour cells (CTCs) are unique cells that originate from the main tumor site. They circulate in the bloodstream, and are implicated in metastasis, immune evasion and recurrence in various cancers. Associated biomarkers of importance for CTC detection include epithelial cell adhesion molecule (EpCAM), human epidermal growth factor receptor 2 (HER2), programmed death ligand-1 (PD-L1), cluster of differentiation 45 (CD45) and other cancer-specific biomolecules. Their roles as standalone biomarkers, have been thoroughly examined in CTC detection, isolation and targeting.

**Methods:**

This review collates key findings on CTC characteristics and biomarker identification. The most recent CTC isolation and detection technologies are discussed, along with individual approaches based on inclusion and exclusion of cell-specific biomarkers. Emerging treatments integrating CTCs, including nanocarrier-mediated drug delivery, have been analyzed. We have discussed both the physical and research barriers in the current landscape.

**Results:**

Recent advances have determined that such biomarkers are more reliable when associated with secondary biomarkers, due to concerns regarding immune evasion and low sensitivity. The identification of these molecules has fast-tracked the development of several groundbreaking technologies.

**Conclusion:**

The prognostic and predictive role of CTCs in various cancers revealed promising results. The development of integrative therapeutics can enhance patient survival and quality of life. These advancements depend on addressing key issues, such as molecular characterization and low abundance of CTCs.

## Introduction

Circulating tumor cells (CTCs) are tumor cells that eroded into the vasculature from the primary tumor and have the capacity to form new cancers away from the original site (Ref. 1). Thomas Ashworth first identified them in 1869, and he described them as having significant metastatic potential (Ref. 2). As our understanding of cancer progressed in the later 20^th^ century, the divergent roles of cells within a tumor were also extensively studied. Tumours may be solid, as in the case of carcinomas, lymphomas and sarcomas, or ‘liquid’, as in the case of leukemias or myelomas (Ref. 3). Thus, tumor pathology has lent itself to be a core discipline in understanding the true nature of cancer, and thereby addressing the questions of how and why it may spread in the human body. The earliest studies, conducted in the 1880s by Rudolf Virchow, established malignancy as the fundamental difference between a benign mass and a malignant mass (Ref. 4). These findings, although valid, were delayed in being substantiated due to a lack of technological advancements. However, with improvements to microscopy, cytology and scientific procedure, earlier hypotheses, like those of Virchow’s, were validated, along with new theories emerging as to the true nature of the cells that contribute to cancerous behaviours. A landmark 1955 study by the Danish scientist, H.C. Engell, suggested that circulating tumor cells could be implicated in recurrence and metastasis, thereby having a direct effect on prognosis, of which the latter conclusion was corroborated by Roberts et al., in 1958 (Refs. 5, 6). Such studies progressed late into the 20th century, with research on the topic continuing well into the 1970s, when Fidler et. al., established that distant metastasis is directly correlated with the number of CTCs (embolic cells) injected in mice (Ref. 7). While establishing CTC roles in the pathophysiology of cancer is important, the primary focus of related research in the 21st century remains the isolation, detection and capturing of CTCs for prognostic, diagnostic and therapeutic purposes (Ref. 8). The concurrent advancements in related technologies, such as microfluidics, liquid biopsies, immunocapture, etc., have lent themselves to developing high-precision techniques that can isolate CTCs (Ref. 8). Despite their low concentration in the bloodstream, their potential as a therapeutic target cannot be ignored, owing to their distinct role in distant metastasis and recurrence (Ref. 9). These techniques span a wide variety of dependent characteristics, as they can be based off the physical characteristic of CTCs, such as size differences between CTCs and normal blood cells, or biomarker differences, such as the presence of EpCAM (Refs. 10–12). However, the primary question remains – Are CTCs the optimal target for preventing or predicting metastasis and recurrence? To answer this question, we must consider the available targets – in the case of solid tumours, the primary targets for treatment or diagnosis are the primary tumor, immune cells in the TME and CTCs. Resection of the primary tumor, especially in the early stages of cancer, is often the standard of care, although it is not always foolproof (Ref. 13). However, complete resection is not a viable option at advanced stages or in certain cancers like those of the lung, owing to the risk in damaging vital organs, as outlined in the NCCN treatment guidelines (Ref. 14). Even in the case of blood cancers, chemotherapy and radiotherapy may drastically improve the patient’s chances of survival, but the QoL is drastically reduced post treatment (Refs. 15,16). Such instances prompt extensive research into CTCs, as their presence can be definitively correlated with malignant cancer, and targeting them would reduce the risk of recurrence at a later stage, which was an issue Virchow highlighted as early as 1888, when Kaiser Wilhelm III died from recurrent laryngeal cancer (Ref. 17).

Thus, in this paper, we examine the modern technologies at the forefront of CTC detection, capture isolation and targeting, with contextual explanations regarding their production. We also discuss their role in altering immune surveillance within the TME, which serves as the basis for highly precise techniques, such as immunocapture, light- and sound-based therapies and nanotechnology.

## Fundamental biology of circulating tumour cells

### Basic properties and origin of CTCs

During metastasis to a distant site, mostly through circulating blood, tumor cells often disseminate by executing a coordinated cascade of intravasation, translocation through the circulation, extravasation and finally, colonization of distant tissues (Ref. 18). These events require a coordinated change in adhesion, cytoskeletal organization and stromal signalling that enable vascular entry (Ref. 19). Once tumour cells breach the endothelial barrier and enter the vasculature, they are classified as CTCs (Ref. 20).

The circulatory environment is highly hostile to these CTCs, as they are exposed to shear stress, oxidative damage and continuous immune surveillance (Ref. 21). As a result, only a very small fraction of intravasated cells survives long enough to contribute to metastatic spread. To endure these conditions, CTCs depend on strategies such as modulation of biomechanical and cytoskeletal properties to better tolerate hemodynamic forces during circulation (Ref. 22). They also activate stress–response pathways and undergo metabolic remodelling that enhances their ability to withstand the conditions in the bloodstream (Ref. 23). To avoid immune surveillance, CTCs frequently engage in transient interactions with platelets and immune cells, which provide shielding and modulate immune detection (Refs. 21,24), this is discussed further in Section “Mechanism to escape immune surveillance”.

CTCs can be found in two main distributions, either as single cells or clusters. The functional and distinctions and clinical relevance of CTC clusters are covered in Section “Biology and Clinical Role of CTC Clusters”. Similarly, the stemness-associated and epithelial-mesenchymal plasticity programs that influence CTC survival and metastatic potential are discussed in Section “Stemness and Epithelial Mesenchymal Transition in CTCs”.

Tumour cells detach from the primary tumour and enter the bloodstream through intravasation. Upon entering circulation, CTCs encounter a highly hostile environment that limits their survival and metastatic potential. Loss of attachment to the ECM can trigger anoikis, which can lead to apoptosis. In parallel, CTCs are also exposed to shear stress in the bloodstream, which can result in cellular fragmentation and death. Immune surveillance further eliminates CTCs through cytokine-mediated cytotoxicity and interactions with immune cells. Despite these challenges, a subset of CTCs survives circulation and metastasizes in distant organs, leading to metastatic tumours.

### Stemness and epithelial–mesenchymal transition in CTCs

CTCs comprise a highly heterogenous population, which may include tumour cells, or drivers of proliferating cells, i.e. cancer stem cells (CSCs) (Ref. 25). These cells possess unique abilities that enable them to separate from the primary tumour, enter the bloodstream and initiate metastasis at distinct locations (Ref. 26). Introducing CSC biology in the context of CTCs is therefore essential, as accumulating evidence suggests that only a subset of CTCs have true metastatic capabilities (Refs. 27, 28).

Two hypotheses are used to explain the origin of circulating CSCs (CCSCs). The first hypothesis suggests that CCSCs originate within the primary tumour. The second hypothesis proposes that CCSCs may arise from a state of dormancy after evading the primary tumours and subsequently propagate to form new cancers. These cells must navigate through challenges such as escaping the immune surveillance system and surviving in the hostile host environment. The subset of circulating tumour cells that successfully survives exhibits complex cascades of metastasis (Refs. 29, 30).

A connection can be assumed between CTCs and CSCs due to the shared properties between the two regarding self-renewal, differentiation, maturation and resilience to apoptosis. CCSCs contribute to tumour heterogeneity, metastasis and therapeutic resistance. The sequential steps involve complex phenotypic plasticity, as well as epigenetic and genetic factors (Refs. 1, 31). Consistent with this, stem-like CTCs have been detected in multiple cancer types, including breast, lung, glioblastoma and colorectal cancer (Refs. 32–35), using CSC-associated markers such as CD44^+^/CD24^-^, ALDH1^+^, CD133 (Ref. 36) and pluripotency-related transcription factors including OCT4, NANOG and SOX2 (Ref. 37). Clinically, the presence of these stem-like CTCs correlates with increased metastatic potential, therapy resistance, disease recurrence and poor prognosis (Refs. 38, 39) .

Epithelial-mesenchymal transition (EMT), a progressive process commonly observed across various cancers, has been linked to the properties of CSCs. EMT often leads to the development of mesenchymal-like CTCs, these CTCs exist as a singular unit, while partial EMT is often associated with multicellular CTCs. This suggests that the regulation of stemness may differ between individual CTCs and those that are found in clusters, highlighting the necessity for therapeutic strategies that specifically target the physical state of CTCs in circulation (Refs. 40, 41). Importantly, EMT is not a binary process, and many CTCs display hybrid epithelial/mesenchymal (E/M) phenotypes that combine both epithelial survival advantages with mesenchymal invasive traits. Emerging evidence also suggests that these cells can lead collective invasion and coordinate multicellular dissemination, all while retaining their self-renewing capacity and plasticity (Ref. 42). Hybrid E/M CTCs are recognized as the most aggressive and plastic subpopulation, exhibiting strong self-renewal capacity and metastatic competence (Refs. 43, 44).

### Biology and clinical role of CTC clusters

Although most CTCs are single cells, a small portion circulate as a group of clustered cells, this has shown to be an adaptive mechanism that improves the survival of CTCs in the circulatory system, while also enhancing their metastatic capabilities (Refs. 45, 46). CTCs have traditionally been defined as groups of tumour cells with two nuclei that have the ability to shed as clusters from the primary tumour, formation through cellular aggregation has also been observed. These clusters can be composed of tumour cell-tumour cell homotypic clusters or they can be composed of tumour cell-blood cell heterotypic clusters (Ref. 45).

#### Homotypic clusters

Homotypic clusters consist exclusively of tumour cells that disseminate as cohesive units from the primary tumour or arise through intracellular aggregation in the bloodstream. Compared to singular CTCs, homotypic clusters exhibit a marked uptick in metastatic efficiency, this has been attributed to cell-cell interactions that promote tumorigenic properties (Refs. 46, 47). Their formation is driven by tumour cell-cell adhesion mediated by adhesion and junction molecules such as CD44, intracellular adhesion molecule 1 (ICAM1) and plakoglobin, which reinforce cluster strength under stressful conditions, such as intratumour hypoxia (Refs. 45, 48). At the molecular level, homotypic clustering is associated with transcriptional and epigenetic programs that promote stemness, proliferation and survival. This is especially done through upregulation and DNA hypomethylation of key stemness genes such as OCT4, SOX2 and NANOG (Ref. 49), as well as activation of the EGFR and PAK2/FAK signalling pathways (Refs. 46, 50). In addition, homotypic clusters had increased expression of keratin 14, which showed lower major histocompatibility complex 2 (MHC2) gene expression, suggesting an adhesion and immune evasion phenotype for clusters (Refs. 45, 51). A promising target for the mediation of homotypic clusters is heparanase, an extracellular matrix-degrading enzyme. Overexpression of heparanase has increased CTC clustering and induced aggregation of ICAM1 and FAK in the bloodstream (Ref. 52). A phase I trial evaluated the drug digoxin for its ability to disrupt heparanase-associated survival of CTC clusters (Ref. 53), this is discussed further in Section “Small Molecule Therapeutics”.

#### Heterotypic clusters

Heterotypic clusters involve clustering with cell types such as WBCs, specifically neutrophils and myeloid-derived suppressor cells, cancer-associated fibroblasts have also been found to form clusters (Refs. 45, 54, 55). Sequencing analysis revealed that CTC-neutrophil clusters are the most common (Ref. 54). The existence of cytoskeletal bridges between tumour cells and other cells most often influences the formation of clusters. Key cytoskeleton genes, such as TUB, GLU and VLM, were observed to be highly upregulated in metastatic breast cancer (Ref. 56).

Patients with CTC-WBC clusters have significantly worse overall survival after 6 months of treatment than patients without CTC-WBC clusters in their blood (Ref. 57). Heterotypic clusters have been found to use their clustered partner for protection, while also enhancing dissemination and metastatic growth. For example, CTC-neutrophil clusters have been found to have enhanced cell-cycle progression transcriptomes (Ref. 54). Similarly, cancer-associated fibroblasts have been found to aid in the migration or circulation of the tumour cells (Ref. 58). Finally, myeloid-derived suppressor cells were found to contribute to metastatic efficiency through promotion of CTC proliferation and survival through the ROS/Notch/Nodal pathway (Ref. 55). While certain heterotypic links have been uncovered, further research is required to fully understand their roles in cancer progression.

## Mechanism to escape immune surveillance

Escape from the immune system has long been seen as a crucial step in the development of tumours and their progression (Ref. 59). The survival of CTCs in the bloodstream also has clinical applications as it allows for the early detection of tumours in patients. This, in turn, allows clinicians to determine the risk of tumour relapse after surgical removal, and any possible resistance to treatments (Ref. 60).

Cancer cells undergo metastatic dissemination and dormancy (Ref. 61). The communication between inactive cancer cells and extravasation sites involves different immune and stromal cells (Ref. 62). After exiting the tumour microenvironment (TME), CTCs are exposed to vigorous immuno-surveillance in non-cancerous tissues. Due to the volume disparity between resident immune cells and CTCs outside the immunosuppressive environment of the tumour, CTCs are easily disintegrated by tumour targeting immune cells. Although the circulatory system represents a hostile environment for tumour-derived cells, due to the abundance of peripheral immune cells, a small subset of CTCs can still survive circulation and metastasize to distant sites (Ref. 59).

The expression of major histocompatibility complex 1 (MHC 1) is inhibited by tumour cells to escape cytotoxic T lymphocytes (CTLs), thereby triggering the activation of natural killer (NK) cells (Ref. 63). CTCs evade immune supervision by attaining a ’pseudo normal’ phenotype over the transfer of platelet-derived MHC I molecules and the intervention of cytokeratins with MHC I interactions (Ref. 64).

In non-classic MHC molecules, human leukocyte antigen (HLA), like HLA-G, functions by binding to immune cell receptors such as Killer cell immunoglobulin-like receptors (KIRs), CD8 and LIR-1, leading to protection against immune-cytotoxicity mediated by T cells and NK cells (Ref. 59). Soluble HLA-G (sHLA-G) is generated through alternative splicing and acts as a systemic modulator of antitumor responses. High levels of sHLA-G, both free and vesicle-bound, are connected with poor outcomes in clinical settings in breast cancer patients, indicating immune evasion and CTC survival (Ref. 65).

The Fas receptor (CD95) is present on many immune cell types. The physiological ligand, FASL binds to the FAS receptor, triggering a signalling cascade within the cell that eventually leads to apoptosis (Refs. 66,67). The tumour cells that express FASL can actively induce apoptosis in immune cells that would recognize and destroy them, thereby evading immune surveillance and clearance. Tumour cells undergo downregulation of FAS expression while upregulating FASL expression (Ref. 68). Loss of FAS decreases the vulnerability of tumour cells to death induced by immune cells, weakening their chances of being eliminated. When FASL is upregulated on tumour cells, it improves their ability to activate apoptosis in immune cells, thereby suppressing immune responses against the tumour and CTCs (Ref. 59).

CD8+ T cells can be kept in an inactive state by CTCs by expressing immune checkpoint proteins. Most research currently focuses on the interactions between these proteins. The ideal example includes the interaction between programmed death protein 1 (PD1) and its associated ligand (PD-L1). The PD-1-PD-L1 interaction maintains cell survival by reducing cytokine production and simultaneously suppressing T-cell proliferation (Ref. 69). However, CTCs from various cancers have the ability to express PD-L1, making it a poor biomarker. Additionally, the heterogeneity regarding PD-L1 expression in CTCs when compared to primary tumours might provide an advantage in immune evasion (Ref. 70).

Once cancer cells disseminate, CTCs rely on interactions with hemopoietic cells like platelets, neutrophils, monocytes and T_reg_ cells for their survival (Refs. 21, 24). Platelets encapsulate CTCs, sheltering them from the body’s immune response, while also protecting them from the excess shear stress exerted by blood flow (Refs. 71, 72). The induction of platelet aggregation by tumour cells can facilitate the process of extravasation and adhesion, in addition to immune response evasion (Ref. 73). The release of transforming growth factor-β (TGF-β) by platelets can deactivate NK cells, while the transfer of MHC-I complex from granular platelets to CTCs plays a crucial role in protecting CTCs against the cytotoxic assault of NK cells (Ref. 74)

## Techniques for enriching, detecting and isolating CTCs

### Enrichment of CTCs

Numerous methods have been developed with the goal of CTC capture, however due to the rarity of CTCs in blood samples, it still proves challenging to isolate CTCs from the bloodstream accurately (Ref. 1). CTCs can be separated based on different methods such as immunoaffinity, physical methods and biological methods (Refs. 1, 75, 76). Currently, the only Food and Drug Administration (FDA) approved enumeration technique is the immunoaffinity-based CellSearch (Ref. 77). However, the Parsortix system received FDA clearance in 2022 for the capture and harvest of CTCs, expanding the range of clinically validated technologies.

#### Immunoaffinity-based methods

Immunoaffinity methods were amongst the first isolation techniques developed for CTC enrichment. The basis of this technique is unique as it uses specific antibodies designed to target antigens expressed exclusively on the surfaces of CTCs. Enrichment approaches can be broadly classified as positive and negative selection. Positive enrichment isolates CTCs by directly targeting tumour-associated markers, such as EpCAM, epidermal growth factor receptor (EGFR), HER2, mucin 1 (MUC1) and N-cadherin (Refs. 78, 79). In contrast, negative enrichment removes hematopoietic cells, primarily by targeting leukocyte makers such as CD45, leaving unlabelled CTCs behind (Ref. 79). An advantage of negative enrichment is that it can retain CTCs that do not express the targeted antigen. (Refs. 75, 80). However, positive enrichment methods generally yield a higher purity.

In practice, immunoaffinity-based enrichment represents a broader methodological framework than a single platform. These approaches differ (i) in selection strategy (positive versus negative enrichment), (ii) antibody presentation, (iii) marker strategy (Ref. 81). While early immunoaffinity methods primarily relied on EpCAM-based positive selection, more recent approaches have introduced combinatorial marker panels to address the heterogeneity of CTCs and epithelial-mesenchymal plasticity (Refs. 81, 82). Platform-specific implementations of immunoaffinity principles, such as immunomagnetic, microfluidic and hybrid systems, are discussed in later sections.

**Figure 1.**
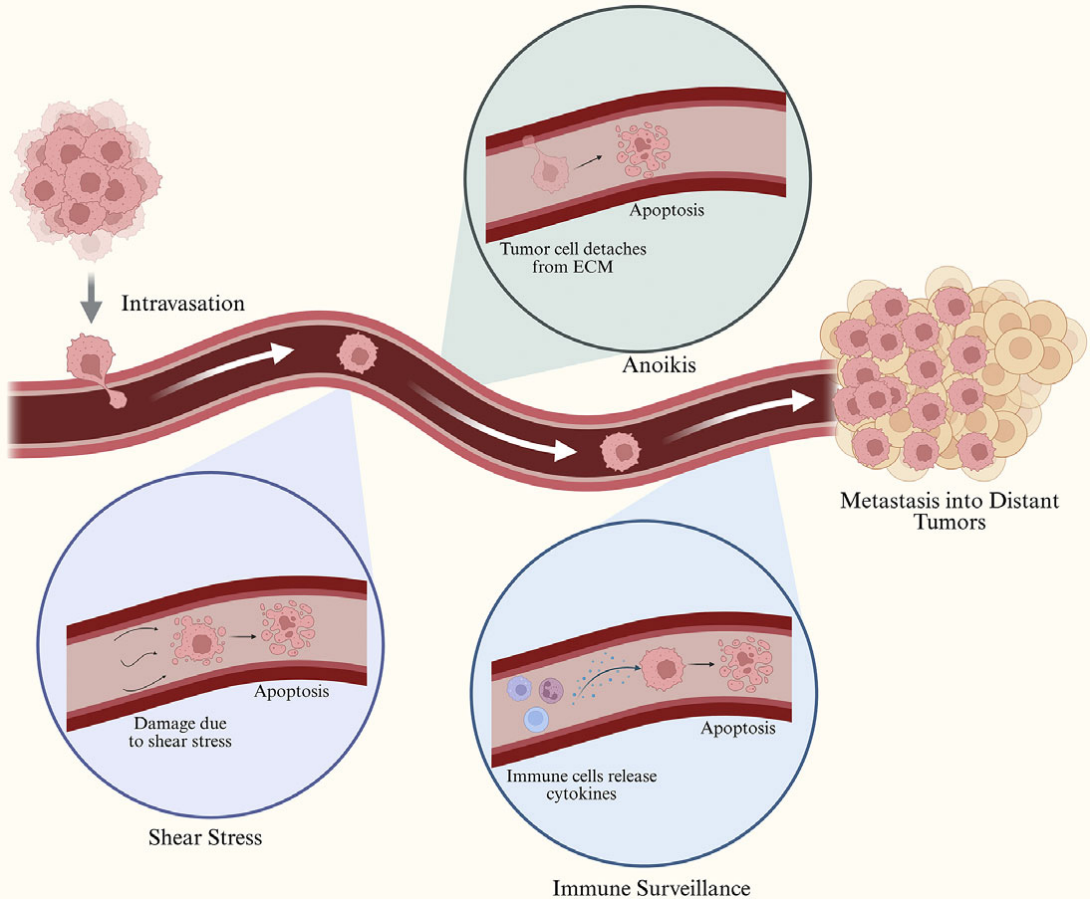
Biological challenges faced by CTCs during hematogenous metastasis.

The most popular example of immunoaffinity-based enrichment is the CellSearch© technology. This system uses whole blood and isolates CTCs based on EpCAM expression through the use of magnetic core particles coated with anti-epithelial cell adhesion molecule (anti-EpCAM) antibodies. Cells that are EpCAM-positive are sorted via magnetic field, and immunostained for CD45 and cytokeratins. Events/samples/cells which express cytokeratins, have a nucleus, do not express CD45, contain a DAPI-stained nucleus and are larger than 4×4μm^2^ in size are classified as CTCs (Refs. 83, 84). Figure 1 details the role of EpCAM in CTC metastasis. (Refs. 83, 84)

EpCAM is highly involved in CTC-driven metastasis, cells that overexpress EpCAM separate from the primary tumour before entering the bloodstream as CTCs. CTCs can either exist as single cells or aggregates, where EpCAM plays an important role in their adhesion. Certain CTCs undergo EMT in the primary tumour leading to the downregulation of EpCAM, allowing for single cells/ loosely aggregated CTCs. Once they reach distant sites, CTCs interact with the stroma, leading to metastasis and the formation of secondary tumours.

#### Enrichment based on physical characteristics

CTC enrichment through physical separation is dependent on the differences in the density, deformability, electrical properties and size between blood cells and CTCs (Ref. 1). Size isolation through an 8μm diameter polycarbonate TRACK-ETCH membrane was found to have low efficiency. However, this method has been improved through the introduction of a flexible micro spring array device, which incorporates a pressure-regulating system. This device is able to achieve 90% detection in 76% of samples (Ref. 85).

Building on this principle, isolation by size of epithelial tumour cells (ISET) was introduced as a standardized, label-free enrichment platform. In the original study by Vona et al., ISET employed track-etched polycarbonate microfilters with 8 μm pores to enrich CTCs from hepatocellular carcinoma patient blood samples; here, only 0.0002% of leukocytes were retained while allowing for detection of cytokeratin-positive CTCs (Ref. 86). Subsequent clinical studies highlight substantial discrepancies between EpCAM-dependent and size-based CTC enrichment approaches. In metastatic breast, lung and prostate cancers, comparison of CellSearch and ISET revealed concordant CTC detection in 55%, 20% and 60% of cases, respectively. These results suggest that technologies limited to CTC capture from EpCAM-positive cells might show poor performance in some cancers, such as metastatic lung carcinoma (Ref. 87). Similarly, in pancreatic cancer, ISET detected CTCs in a significantly higher percentage of patients in CellSearch (93% vs 40%) and yielded higher CTC counts overall (Ref. 88). Other filtration methods include MetaCell and Parsotix, both of which are in use (Refs. 89, 90). MetaCell is an alternative size-based filtration system to isolate CTCs. It uses a porous polycarbonate membrane with a diameter of 8μm, which allows smaller blood components to pass through the membrane while CTCs are entrapped in the membrane. Due to its label-free nature, MetaCell is especially effective in filtering heterogenous CTC populations (Refs. 76, 91). In optimization studies using colorectal cancer cell lines spiked in healthy blood samples, MetaCell demonstrated consistently high CTC recovery rates (≥ 85%) across a range of concentrations, comparable to those reported for CellSearch, and simultaneously, it also achieved ≥ 95% leukocyte depletion. Clinical application of MetaCell showed CTC detection in approximately half of the samples, similar to results seen in ISET, with smaller CTCs possibly limiting sensitivity in certain tumours (Ref. 91). In contrast, Parsortix uses a microfluidic cassette with a tapering channel, which traps larger blood components such as CTCs, while smaller, more flexible cells, such as RBCs, are able to flow through (Ref. 92). This design reduces clogging, facilitates gentle cell recovery and preserves cell viability. Comparative studies demonstrated that Parsortix captures CTCs at rates comparable to or exceeding EpCAM-based platforms, such as CellSearch, including CTCs with low or absent EpCAM expression. However, partial size overlap between leukocytes and CTCs may lead to smaller CTC loss, and leukocyte contamination (Ref. 93).

Beyond filtration-based approaches, other physical property-based methods have also been developed. The Apostream© system uses a dielectrophoresis-based microfluidic system to isolate CTCs by exploiting the difference in the dielectric properties of blood cells and CTCs. Using frequency-dependent electric fields, CTCs experience positive dielectric forces and are deflected toward collection regions, while most blood cells are repelled, which allows for effective separation without antibody labelling (Refs. 93, 94). Apostream demonstrated reproducible recovery percentages between 70 and 75% in spike-in experiments, with there being a strong correlation between input and recovered CTCs. Importantly, the separation method had a post-isolation cell viability of > 95%, allowing recovered cells to be analysed downstream (Ref. 93). However, Apostream requires a large volume of blood, which may limit its clinical use (Ref. 93). Oncoquick© is dependent on differences in density, which allows RBCs and WBCs to be filtered out (Ref. 95). The platform combines density-gradient separation with a porous barrier that stabilizes the layer formation, thereby enriching CTCs together with mononuclear cells, such as leukocytes, marking it as an improvement over Ficoll-based methods. Early clinical and spiking studies showed improved CTC recovery using Oncoquick, with recovery rates in the range of 80-87%, supporting its utility for CTC enumeration. However, the similar density gradients of CTCs and leukocytes often result in substantial co-enrichment, limiting its suitability as the sole enrichment strategy for downstream analysis of CTCs (Ref. 96).

#### Enrichment based on biological characteristics

Detection approaches based on biological characteristics are primarily dependent on the presence of EpCAM, mesenchymal markers and antigen-antibody interactions. Among these, EpCAM-based positive selection techniques, such as CellSearch are commonly used by researchers (Ref. 1). Magnetic nanoparticles covered with a layer of antifouling hydrogel that inhibits nonspecific cell adhesion can be used for CTC capture. EpCAM antibodies are grafted on the hydrogel layer surface, providing greater specificity for CTCs. When tested on mimic clinical blood, results showed that 96% of CTCs were captured (Ref. 97). An aptamer-triggered-clamped hybridization chain reaction (atcHCR), has been used for cloaking/uncloaking and in situ hybridization of CTCs through the use of porous hydrogels. CTCs are captured by the EpCAM and are unharmed due to the gentle release of the hydrogel (Ref. 98). Currently, there is a shortage of universal markers that can be used. For example, CTCs that undergo EMT are often devoid of EpCAM, in which case negative selection methods such as CD45 depletion are more optimal (Ref. 99). RosetteSep is a widely used negative selection-based enrichment method that enables the isolation of both singular CTCs and CTC clusters, while preserving their biological properties. The method relies on the removal of CD45^+^ cells using tetrameric antibody complexes, followed by density gradient centrifugation using Ficoll-Paque. It is a popular negative selection method, as it obtains both CTC clusters and single-cell CTCs. It does so through the removal of CD45-positive cells bound to tetrameric antibodies, before using centrifugation to precipitate them in a Ficoll-Paque density gradient (Ref. 100). The RosetteSep method has been successfully used across multiple cancer types, such as breast, prostate, small-cell lung and gastroesophageal cancer, to isolate intact CTCs and CTC clusters for downstream molecular and functional analyses, including transcriptomic profiling and *in vivo* metastasis assays (Refs. 80, 101–103). However, the method requires a significant amount of time and can prove to be labour-intensive, due to which the reproducibility of results becomes unproductively laborious (Ref. 100).

### Detection methods

#### In vivo detection methods

*In vivo* detection methods have enhanced sensitivity and are mostly used for early detection of CTCs. Presently, it is used only in basic research as it is lengthy, tedious, invasive and expensive. Liquid biopsy and CTC chip implantation methods are used to implement *in vivo* procedures. CTCs break away from the primary tumour and enter extracellular vesicles and the bloodstream, making it an ideal target for *in vivo* detection methods (Ref. 104).

The proposed method for capturing CTCs *in vivo* involves modifying vein indwelling needles with EpCAM antibodies (Ref. 105) and the structured medical wires that capture EpCAM+ CTCs (Ref. 106). Recently used methods include in vivo flow fluorescence Cytometry (IVFC), Photoacoustic Flow Cytometry (PAFC) and the use of the GILUPI Cell Collector© (Ref. 90).

#### Microfluidic-based detection methods

Microfluidic devices have gained popularity for CTC isolation due to the small sample requirements, low cost and continuous processing (Ref. 107) and their ability to alter the properties of both fluids and particles at the microscale level. Microscale separation allows for the selective CTC isolation without the need for external/moving forces, and this can lead to shortened processing times and cost reduction (Ref. 108). These systems can be based on several principles, including inertial focusing, size-based filtration and surfaces coated with EpCAM antibodies (Ref. 109). The CTC-iChip, the ClearCell® FX system and the herringbone microfluidic chip are a few examples of such devices (Ref. 110).

#### Immunocapture detection methods

Immunocapture detection in CTCs makes use of unusual protein expression and genes that are not present in other components of blood to identify CTCs in a more specific and sensitive alternative to traditional CTC detection methods (Ref. 111). The immunocapture method of isolating CTC can be done via methods such as immunomagnetic and microfluidic-based immunocapture. In microfluidic-based immunocapture, the geometry of the microfluidic device serves to maximize interactions between CTCs and the associated ligands that have been functionalized on the surface of the chip. Thege et al. built a microfluidic device with the purpose of capturing pancreatic CTCs using immunocapture, where a combination of anti- MUC1 and anti-EPCAM antibodies was used. Results showed efficient capture of the CTCs while also extending immunocapture past the use of single markers (Ref. 112). Immunomagnetic capture is carried out by negative or positive selection (Ref. 113).

**Figure 2.**
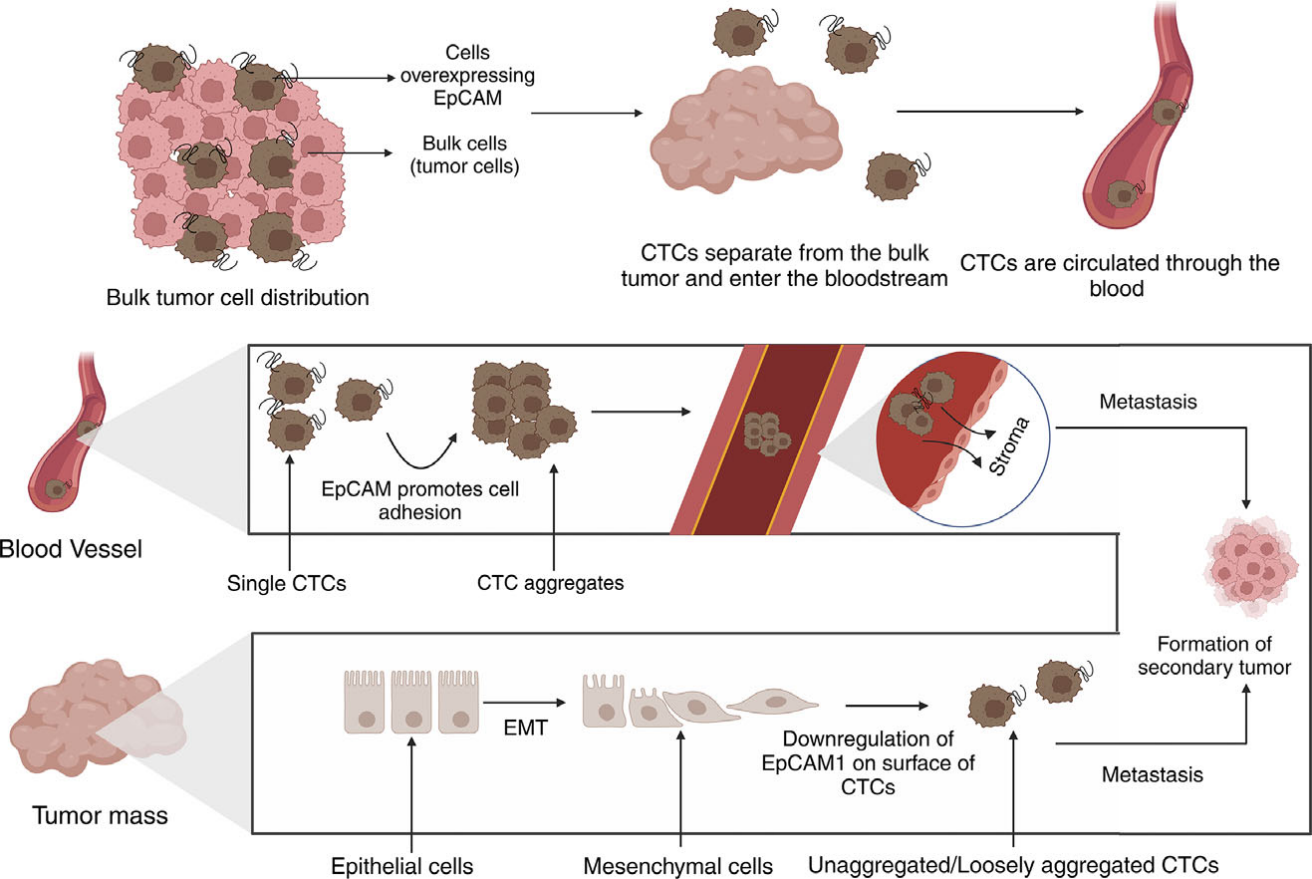
Role of EpCAM in CTC-driven metastasis (Created with BioRender.com).

Positive selection involves coating antibodies that bind to the target cells with magnetic particles, leading to greater sensitivity and specificity. The target cells attach to the antibodies on the magnetic beads when a sample is combined with these particles. The target cells that are bead-bound are then magnetically separated from the remaining sample. Now that the target cells are adhered to the magnetic beads, they can be gathered and subjected to more analysis (Ref. 79). Techniques such as CellSearch©, MACS, Magsweeper and Immunomagnetic separation (IMS) are all used in positive selection (Ref. 113). Figure 2 provides a graphical summary of the positive selection techniques.

**Figure 3.**
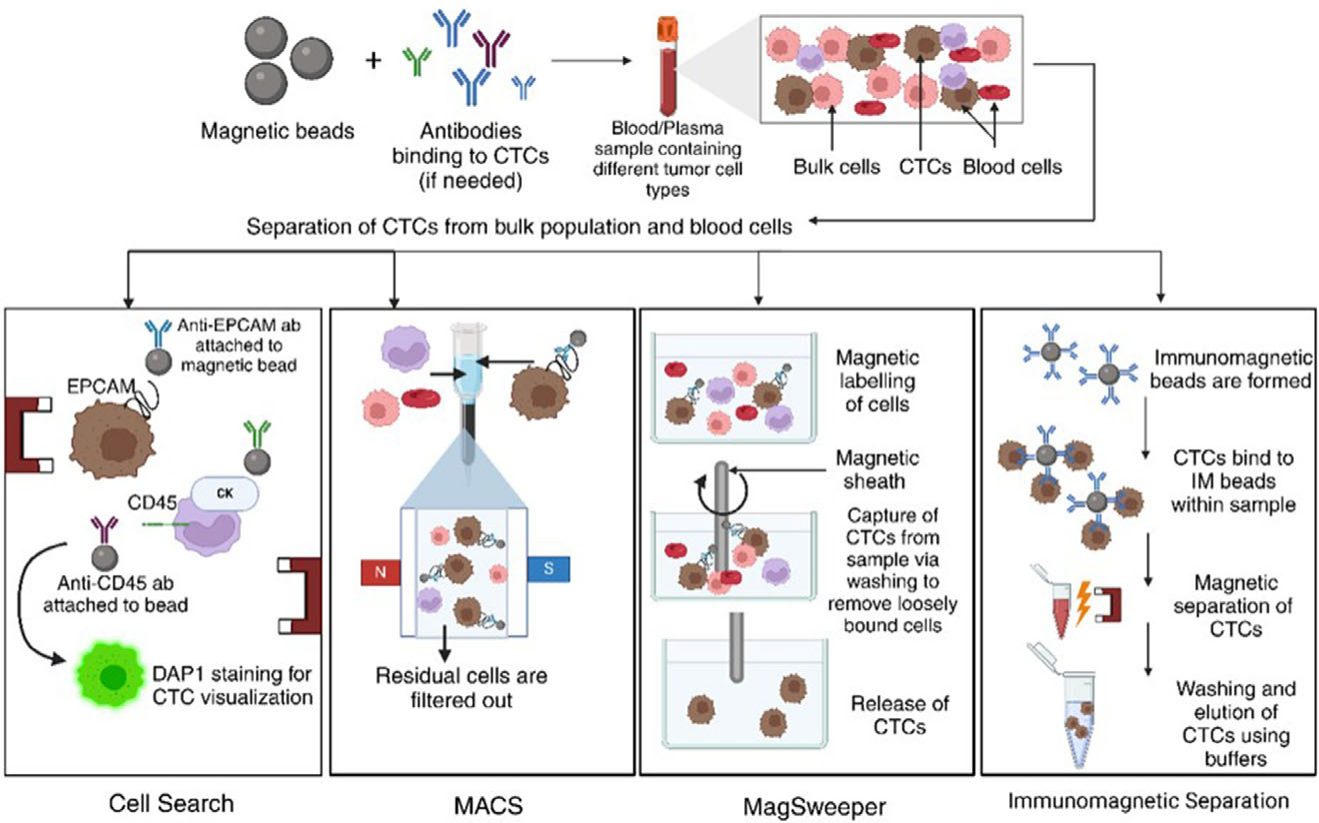
Process overview of different immunomagnetic positive selection techniques for CTC capture. *Note*: ab, antibodies (Created with BioRender.com)

In negative selection, magnetic particles are coated with antibodies that bind to undesirable cell types while sparing the target cell. The undesired cells attach to the magnetic beads when the sample is combined with these particles. The target cells are then left in the supernatant after the undesirable cells that were linked to the beads are removed using a magnet. It is possible to gather the unaltered target cells for later application (Ref. 76), techniques such as EasySep and Rosette Sep have been applied here (Refs. 113, 114). Multiple studies have shown that around 50-70% of patients with metastatic breast, prostate and colon cancers display high levels of CTCs when analysed using immunogenetic assay-based CellSearch® (Ref. 115). Figure 3 summarizes the detection and isolation methods of CTCs. Table 1 describes popular devices employed in the detection/isolation of CTCs alongside their main findings.

**Table 1.** Overview of selected devices and techniques employed in CTC detection and isolation

Method	Principle	Advantages	Disadvantages	Patient application	References
In vitro					
*Size*					
Microcavity Array (MCA)	Micro sieve-like structure with uniform pores, these pores are engineered to match the size differences between cells (6–10 μm), CTCs are usually (15–25 μm) and less deformable than hemopoietic cells. Whole blood is flowed through the pores, with CTCs getting retained on the surface.	Can be performed quickly in a daily routine Only requires blood samples, which are easily obtained No serious side effects	Cell adhesion limits release efficiency of CTCs Clogging of hemopoietic cells may lead to reduced performance	Rates of HCC cell line recovery were high in the blood of healthy volunteers, with larger numbers of CTCs being detected in progressive HCC patients.	(Refs. 118, 119)
Parsortix	Step-based microfluidic cassette with narrowing channels. CTCs, being larger (15–25 μm) and less deformable than hematopoietic cells, are physically trapped at the narrowest step, while smaller hemopoietic cells pass through. Whole blood is flowed through the cassette.	Captures CTC clusters Allows for on-chip staining of cells	On-chip imaging is difficult Slow processing time	Mean capture rate of 62.4% was observed across breast cancer and non-small cell lung cancer, using the 6.5 μm cassette	(Ref. 120)
Parallel Multi-Orifice Flow Fractionation (P-MOFF)	Microfluidic device with alternating contraction channels (60 × 150 μm) and expansion chambers (300 × 300 μm). Particle separation is governed by inertial lift forces, described by the particle Reynolds number (Rep). Larger, less deformable CTCs experience stronger inertial forces and migrate differently through the orifice array, while smaller hematopoietic cells follow the fluid streamlines	Label-free isolation Allows continuous, high-throughput separation of cells without clogging due to hydrophoresis.	RBC lysis and Ficoll density centrifugation required	91.60% of MDA-MB–231 cells and 93.75% of MCF–7 cells were separated successfully, while 90.8% of WBCs were eliminated, confirming that the device can isolate both EpCAM negative and positive CTCs	(Ref. 121)
ClearCell FX	ClearCell FX uses Dean drag force to separate larger cells from smaller cells as cells flow in a spirally wound microfluidic channel	Single-step process, therefore quick processing time High-recovery rates Label-free isolation	May be inefficient for tumours with smaller tumour cells Potential for leukocyte contamination as large population of CTCs overlaps in size with leukocytes	ClearCell allows for sensitivity of 80.4% and 85.7% specificity by ROC curve analysis (cut-off = 3.5 CTCs) in detecting CTCs in 77 clinical samples (56 cancer patients and 21 healthy) using cytokeratin/EpCAM as inclusion markers, and CD45 as an exclusion marker. Sequencing was done on hepatocellular carcinoma samples due to its high counts of CTC, in which they could detect somatic missense mutations in oestrogen receptor (ESR1) and TP53	(Ref. 122)
*Density*					
OncoQuick	OncoQuick enrichment is built on the fact that CTCs have a lighter buoyan*t* density than peripheral blood mononuclear cells (PBMCs), so that they remain at the top of the liquid	Recovery of heterogenous CTCs Up to 25mL of blood can be processed at once	Results have shown high variation in recovery rates (25%–80%)	A comparison with CellSearch in processing blood from 61 patients with multiple different types of cancer, showed that OncoQuick detected at least 1 CTC in only 23% (14 of 61) of patients, compared to 54% (33 of 61) of patients through CellSearch	(Refs. 113, 123)
AccuCyte	Density-based separation system using a lozenge-shaped float that positions at the plasma–blood cell interface during centrifugation. The buffy coat is displaced into a transfer tube with high-density retrieval fluid, then spread onto slides (CyteSpreader) for immunohistochemistry. CTCs are enriched within the buffy coat while avoiding washing steps that cause cell loss.	High recovery rates Associated imaging and mechanical selection tools for CTCs	Can cause cellular damage	Average CTC count was 5 circulating prostate cancer cells/7.5 mL (range, 0–20)	(Refs. 113, 124)
*Membrane filtration*					
Flexible Microspring Array (FMSA)	Contains round-shaped pores with a diameter of 8 μm, flexible micro spring structures which are carved into the parylene filter. The microspring is connected to a pressure regulation system, which passes blood gently through the system, maximising CTC viability with a reverse pressure applied to recover cells from the filter	Rapid processing speed Greater maximal blood volume capacity compared to the standard 7.5 mL No damage to cells	Clogging of filter is highly likely	FMSA device enriched tumour cells with 90% capture efficiency, higher than 10^4^ enrichment, and better than 80% viability from 7.5-mL whole blood samples in <10 min on a 0.5-cm^2^ device. The FMSA detected at least 1 CTC in 16 out of 21 clinical samples (approximately 76%)	(Ref. 85)
Isolation by Size of Epithelial Tumour Cells (ISET)	Diluted blood is passed through a membrane with calibrated pores, usually 8 μm. Once loaded, the blood is filtered by applying a vacuum, resulting in gentle aspiration. Hemopoietic cells usually pass through the membrane pores, with larger CTCs getting trapped.	Easy to scale up for large-scale clinical detection due to simplicity of operation High homogeneity	Slow processing time Diluted blood to avoid clogging of the membrane	ISET and CellSearch comparison in 60 patients with metastatic prostate, lung and, breast cancer. Concordant results were observed in only 55% (11 of 20) of breast, 60% (12 of 20) of prostate and 20% (4 of 20) of lung cancer patients. ISET showed better results in detecting CTCs in non-small cell lung cancer, pancreatic, oesophageal and prostate cancer	(Ref. 87)
Fluid Assisted Separation Technology (FAST)	Introduces an aqueous phase is placed below the chamber, aiding in combating filter clogging. The filter contains pores of 8 μm, with the aqueous phase below the membrane allowing it to diffuse uniformly through the membrane. The final step, centrifugation, isolates and retains CTCs on the filter	Quick processing time Easy and cheap to produce Recovery of heterogenous CTC populations	Low throughput Can only process low amount of blood at a time	The device has been tested on breast, gastric and lung cancer, with a wide range of EpCAM expressions. Clinical detection rates of 83.3% was observed in breast cancer, 82.9% was observed in stomach cancer and 68.6% in lung cancer.	(Ref. 125)
ScreenCell	Requires RBC lysis, however claims 100% cell recovery. Contains filters of different pore sizes (6.5 μm and 7.5 μm) randomly distributed for fixed and live cells. Following filtration, filters are removed by pushing a rod into the device to release the CTCs on the filter.	Quick processing time Quick and easy to produce RBC lysis stage claims 100% recovery of cells	Pores that are uneven or fused can reduce efficiency of capture Prone to clogging, blood must be diluted before loading	Isolated CTCs from 10 patients (five males and five females) with primary lung cancer and CTCs were detected in 80% of patients prior to surgical resection	(Refs. 126, 127)
Microcavity Array (MCA) Filter	Contains a polymethyl methacrylate (PMMA) filtration cartridge with a filter consisting of silver and gold. There is a total of 10,000 cartridges arranged in 100 × 100 array. Cavities have a diameter of 8–11 μm and are spaced 60 μm apart, with a negative pressure applied to the device resulting in CTC capture.	Allows for on-chip staining Up to 4 samples can be processed at once	Low throughput	Direct comparison with CellSearch identified 77% (17/22) of patients as CTC positive using MCA filter and 32% (7/22) using CellSearch. With matched patients, the MCA filter detected a median of 13 CTCs (range 0–291, 7.5 mL) and 0 CTCs (range 0–37, 7.5mL) using CellSearch	(Refs. 128, 129)
*Dielectrophoresis*					
ApoStream	Device requires initial Ficoll separation step which isolates PBMCs. These cells are then passed over a dielectrophoresis field in a laminar stream, with its alternative current pulling CTCs to the floor of the chamber. After which iCys laser scanning cytometer can be used for CTC enumeration.	Label-free iCys laser scanning cytometer for CTC enumeration Quick processing time	Expensive to synthesize	Device was tested using MDA-MB–231, A549 and ASPS–1 cell lines. Recovery percentages ranged between 55% and 68%, depending on the cell line. 97% of MDA-MB–231 cells were recovered successfully	(Refs. 130, 131)
In Vivo					
*Real time monitoring*					
Cytophone	Cytophone employs PAFC to detect CTCs without a blood draw through sending a near-infrared pulse laser (1060 nm, subnanosecond 10 ns pulses) transcutaneously into hand veins, where melanin in melanoma CTCs and haemoglobin in RBCs generate thermoacoustic waves. These waves are detected using an ultrasound transducer, with RBCs producing baseline signals and CTCs producing sharp peaks	Non-invasive real time monitoring Label-free detection High Sensitivity	Safety concerns regarding repeated laser exposure	Individual CTCs can be detected by the Cytophone at a concentration of ≥1 CTC/ml in 20 s and could also identify CTC clots. in vivo tests were verified using ex vivo. These data suggest the potential of in vivo blood testing with the Cytophone for early melanoma screening	(Ref. 132)
In Vivo Fluorescence Flow Cytometry (IVFC)	IVFC employs in vivo flow cytometry by directing a focused laser beam onto superficial blood vessels, where fluorescently labelled tumour cells passing through the detection zone emit fluorescence signals. These signals are then captured by a confocal detector, enabling real-time monitoring	Non-invasive Dynamic Monitoring High Sensitivity	Requires extrinsic staining of CTCs, which can cause cytotoxicity and photobleaching Current research focuses on superficial tissues, with deeper tissues unamenable to real time mentoring	N/A	(Ref. 133)
GILUPI CellCollector	An anti-EpCAM wire is inserted into the veins of a patient using an intravenous cannula for 30 minutes, which allows for CTC sampling from the peripheral blood of patients	High purity of isolated cells Negligent loss of cells	More invasive for the patient in comparison to blood draw Can only recover EpCAM-positive CTCs	Device was validated in both breast and non-small cell lung cancer patients, where they isolated CTCs from 91.6% (22 of 24) patients from all tumour stages including early stage tumours.	(Ref. 106)
Immunocapture					
*Immunomagnetic*					
*Positive selection*					
CellSearch	CTCs expressing EpCAM are isolated through anti-EpCAM antibodies and magnetic core particles. These CTCs are immunostained with cytokeratins and CD45 for detection	Can process up to 8 samples at once FDA approached Semi-automated	Recovery of EpCAM-positive CTCs only	Recovery rates of ≥85% have been reported, with clinical detection rates of 71.4%, but lower figures than this have also been reported. Groups have suggested that is ranges from 42% to 90% and clinical detection rates between 20% and 77.5%	(Refs. 113, 134)
Magnetic Activated Cell Sorting (MACS)	Whole blood is coated in antibodies against cytokeratins and human epidermal growth factor 2 (HER2), with the most common antibody being EpCAM. The blood is passed through a column with ferromagnetic steel wool, which can process 15mL blood. CTC-bead conjugates are held within the steel wool fibre and can be extracted	Able to process up to 15mL of blood Easy elution of CTCs Mix of antibodies available to increase CTC capture.	EpCAM positive CTCs recovery only MACS system is suited for tissue samples	Poor reproducibility using the system with efficiencies as high as 90% and low as 25% The MACS system has been suggested to work better with tissue samples, with high CTC yields being hard to obtain through blood.	(Refs. 135, 136)
Biomimetic Immunomagnetosome (IMS)	Magnetic nanoclusters are camouflaged as leukocyte membrane fragments, forming an EpCAM-conjugated magnetosome, which has high CTC recognition efficiency.	High purity due to repelling of leukocytes	Has not conclusively been tested on patient samples Recovery of only EpCAM-positive CTCs	N/A	(Ref. 137)
Strep Tag	Whole blood is incubated with strep-tactin-coated magnetic beads (STMBs), which are then conjugated with strep-tag II-derived IgG. Following magnetic enrichment, the addition of d-biotin binds to the STMBs via strep-tactin allowing for easy CTC release	Easy release of CTCs by simple addition of d-biotin Mix of antibodies available for CTC capture	Recovery of only EpCAM-positive CTCs	The system was tested in cancers such as lung, gastric, liver and pancreatic cancer. Comparing with CellSearch with blood samples from five patients, and anti-EpCAM/EGFR/HER2-IgG-STMBs for Strep Tag was performed. Results showed recovery of 12–72 CTCs in 1mL of blood for Strep Tag, and 0–23 CTCs in 7.5mL for CTCs.	(Ref. 138)
*Negative selection*					
RosetteSep	A multitude of antibodies targeting several biomarkers, such as cluster of differentiation 2 (CD2), CD66b, CD19, CD16, CD36, CD45 and glycophorin A. Upon mixing the antibodies, a rosette network of unwanted blood cells is formed, and Ficoll centrifugation allows for the removal of RBCs and leukocytes.	Mix of antibodies allows for highest rate of capture Recovery of heterogenous populations of CTCs	May accidentally remove CTC clusters	Mean rate of recovery is 62.5% when testing spikes human prostate and ovarian cell lines in blood. The device was also able to detect CTCs in the blood of 90% (18/20) of metastatic epithelial ovarian cancer patients and 76.9% (10/13) of prostate cancer patients.	(Ref. 139)
EasySep	Uses a magnetic field to retain leukocytes, while the resulting supernatant retains the heterogeneous population of label-free CTCs since no antigen targeting is involved. The samples take 25 minutes to capture, process and label free viable cell.	Recovery of heterogenous populations of CTCs	Variable recovery rates May accidentally remove CTC clusters	Detection of CTCs in patients is highly variable depending on the epithelial type of cancer. CTCs were detected in 57% of patients (47/84), with a wide range of detection seen between tumours (44% colon, 80% gastric, 100% lung and 50% ovarian	(Refs. 140, 141)
*Microfluidics*					
CTC Chip	The chip contains 78,000 anti-EpCAM-coated micropillars, which provide a large surface area for CTC capture	High sensitivity of CTC detection High viability of cell recovery	Difficult to scale up due to the complex geometry of the chip Slow processing rate	The chip was tested for colorectal, lung, prostate and pancreatic cancer and successfully identified CTCs in 99% (115 of 116) of patients with 50% purity. The CTC chip was also able to isolate CTCs from 100% (7/7) of patients with early-stage prostate cancer	(Refs. 142, 143)
Herringbone (HB) Chip	Designed with eight microchannels which have herringbone grooves on the upper surface of the chip, with the inner walls of the device being functionalized with anti-EpCAM antibodies. Herringbone grooves lead to laminar flow disruption, causing an increased number of interactions between CTCs and antibody coated surfaces	Grooves increase antibody-CTC contact, for greater cell capture	Recovery of EpCAM positive CTCs only	When tested on patients with metastatic prostate cancer, with CTCs being detected in 93% (14/15) of patients, with CTC clusters of 4–12 also being identified.	(Refs. 144, 145)
Graphene Oxide (GO) Chip	The device is composed of a flower-shaped gold pattern over which functionalized GO nanosheets are placed, leading to an increased surface area	Large surface area for greater capture of CTCs Simple chip design	Recovery of EpCAM-positive CTCs only	The chip was tested on a small cohort of metastatic prostate and breast cancer patients, with capture efficiency ranging from 67%–80%	(Refs. 146, 147)
High-Throughput Micro Sampling Unit (HTMSU) Chip	The HTMSU chip has 51 high-aspect microchannels that are functionalized with anti-EpCAM antibodies/anti-PSMA antibodies for CTC capture. The unique aspect of this device is the on-chip single cell conductometric counting which detects the unique properties of single CTCs	On-chip single-cell conductometric counting for CTC enumeration Quick processing	Recovery of EpCAM/PSMA positive CTCs only	High throughput device with 1mL capacity for blood, which is processed in 2.7 minutes. With a >97% capture efficiency from MCF–7 cells with trypsin used to release unlabelled captured CTCs.	(Ref. 148)
Nanovelcro	Principle behind the technology relies on the anti-EpCAM-coated silicon nanowire substrate (SiNS) to immobilize CTCs on the chip. The chip also contains polydimethylsiloxane for laminar flow disruption, thereby increasing contact between CTCs and EpCAMs for capture	4 generations of the chip developed for different clinical utilities 3^rd^ and 4^th^ generation chips adapted for easy use	Recovery of EpCAM-positive CTCs only	The first generation of the chip captured CTCs in 100% (40/40) of patients with prostate cancer. The third-generation chip improved recovery rates and viability of cells, while the fourth-generation chip improved cell release	(Refs. 149, 150)
Isoflux	Blood is input into the device where it incubates with antibody-coated magnetic beads, with up to 4 samples being run in parallel. Blood then flows through the chip, and CTCs are captured in an isolation zone at the top of the device, where they are exposed to an external magnetic field	4 samples can be processed simultaneously Increased EpCAM sensitivity	Biased towards epithelial-like cells	When comparing CellSearch and Isoflux in matching prostate cancer samples, CTC counts (>4 CTCs) were observed in 95% (21/22) of samples when processed with Isoflux, in comparison to 36% (8/22) when processed with CellSearch	(Ref. 145)
CROSS Chip	CTCs can be isolated from unprocessed whole blood using the CROSS Chip, a label-free chip that filters based on their size and deformability. The chip contains anisotropic microfilters with narrow gaps (5 μm wide × 20 μm high). Normal blood cells, which are smaller and more deformable, can squeeze through the pores, while CTCs which have a larger size get trapped in the microfilters	Label-free High capture efficiency No pre-processing required	Low volume capacity Possible risk of leukocyte contamination	Out of 9 patients with colorectal cancer, 7 out of the 9 patients showed ≥ 3 CTCs per 7.5mL using CROSS Chip, while none of the patients showed ≥ 3 CTCs per 7.5mL when using CellSearch	(Ref. 151)
RUBY Chip	The device contains an inlet that guides the sample into multiple filtering chambers. Micropillars are present in each chamber, which allow deformable hemopoietic cells to pass through while trapping CTCs.	High detection rates Short processing times High capture efficiency	Potential clogging Loss of smaller CTCs due to its size-based mechanism	Clinical validation was performed in 18 patients (8 non-metastatic, 5 metastatic treatment-naïve and 5 metastatic progressing under treatment). The average CTC detection was 77.8%, with the average total CTC count being 6.4, 101.8 and 3.2, respectively.	(Ref. 152)

#### Single-cell detection methods

Despite their great versatility, these cell enrichment-based methods still exhibit compromised efficiency in CTC isolation, limiting their detection sensitivity. Moreover, these methods typically rely on dedicated chip fabrication, tedious operations, or costly equipment, restricting their use in a routine diagnostic test. New strategies with high time- or cost-efficiency and single-cell sensitivity are critical for the implementation of CTC detection in the clinic, particularly for cancer screening (Ref. 116).

The DEPArray technology is a microchip-based digital sorter, combining both microfluidic and microelectronic image-based isolation of single CTCs. It operates on the principle of dielectrophoresis, whereby non-uniform alternating electric fields generated by an array of over 300,000 programmable micro-electrodes create stable “DEP cages”, which are capable of trapping and levitating individual cells without physical contact. Cells loaded into the device’s chamber are randomly distributed in these cages and visualized using high-resolution bright field and multi-channel fluorescence microscopy, which enables single-cell level morphological and phenotypic analysis (Ref. 117).

Micromanipulation involves the manual picking of stained CTCs using a microinjector, which is controlled by a micromanipulator, and the whole process is visualized using an inverted microscope. As it is a manual process, it can be time consuming; however it allows for the disassociation of CTC clusters and CTC-WBC clusters into single cells to allow for subsequent single-cell analysis of intra-cluster heterogeneity. Automated adaptations of micromanipulation, such as the CellCollector system, employ high-precision glass micro-capillaries mounted on robotic arms to improve both efficiency and reproductivity, providing a more standardized alternative to fully manual approaches (Ref. 113).

Magnetic beads conjugated with CTC-targeting antibodies are added to blood/plasma samples that contain different tumour cell types. Bound CTCs are then separated using methods such as CellSearch, where anti-EpCAM antibodies are conjugated to magnetic beads, which are then stained with DAPI for easier visualization. In MACS, cells are magnetically labelled and a magnetic sheath is used to filter out the non-residual CTC population. In MagSweeper, CTCs are labelled with magnetic beads, which are then captured by washing to remove loosely bound cells. In immunomagnetic separation, immunomagnetic beads are formed, which bind to CTCs, magnetic separation helps differentiate the CTCs, before elution of purified CTCs using buffers.

## Therapeutic strategies targeting CTCs

CTCs are linked with poor prognosis in multiple cancers, due to their ability to evade immune surveillance and promote metastasis (Ref. 153). Early diagnosis of CTCs, along with their timely elimination, may increase the overall survival (OS) of patients (Ref. 154).

### Conventional approaches

Surgical resection remains the cornerstone for early-stage cancers and can reduce both the possibility of CTC formation and the progression of metastasis. However, in certain cancers, such as hepatocellular carcinoma (HCC), it was observed that surgical resection caused an uptick in CTC populations (Refs. 155–157). Both CTC detection and incidence showed increases postoperatively, with CTC counts increasing in 41.7% of patients, decreasing in 25.2% and remaining unchanged in 33.1% of patients’ post-surgery. Evidence suggests that in early-stage HCC, tumour cells tend to spread through the portal venous system and can be driven into the bloodstream via hepatic vein tumour thrombi during surgery. Refinements, such as the “no-touch” technique, which aims to minimize tumour handling, showed no post-operative CTC increase in 5 patients; however, further investigation is required (Ref. 157). Similarly, Tamminga et al. observed that surgical resection led to paradoxical metastasis; however increase in CTC volume was not observed. Suggesting that surgery may alter CTC biology in ways other than increasing CTC count (Ref. 158).

Chemotherapeutics have been found to reduce the total volume of CTCs, along with preventing early metastasis (Ref. 159). Chemotherapy was found to reduce CTC counts in 15/30 of the patients treated. However, chemotherapy was also found to activate the CTCs’ phenotypic switch through cytotoxic stress, activating partial-EMT and rendering surviving CTCs more resistant to conventional therapies while simultaneously making them more responsive to tumour environmental stimuli, increasing the chances of distant organ metastasis (Refs. 153, 160, 161). Since partial-EMT CTCs show resistance towards conventional chemotherapy, this method was found to result in worse prognoses and lowered treatment efficiency over time (Ref. 153).

### Immunotherapeutic approaches

Many CTCs and other distant tumour cells express surface markers such as PD-L1 and CD47, which assist in escaping immune surveillance, as stated earlier (Ref. 162). These protein-ligand interactions are responsible for the suppression of tumours along with the impairment of an antitumor response. The blocking of PD-L1 makes CTCs more susceptible to immunotherapy, making PD-L1 a favourable therapeutic target (Ref. 163).

CTCs use platelets to withstand shear forces, shield themselves from the immune system and limit the presentation of antigens on CTC surfaces (Ref. 71). Platelets stick to sites of injury sustained by vascular endothelial cells. Here, the platelets release microparticles that promote anti PD-L1 binding to CTCs, effectively blocking PD-L1 on tumour cells and inhibiting metastasis (Ref. 12). Activation of platelets can induce the recruitment of other immune cells, which mount a strong anti-tumour response after the initial PD-L1 blockade in preclinical models (Ref. 164). However, despite promise in preclinical models, clinical trials involving anti-PDL1 have demonstrated a large range of response rates (10-30%) in tumours with no selective biomarker. Patients who have PD-L1 high CTCs before treatment had better rates of response to anti-PD-L1 mAbs as well as longer progression-free survival (PFS) and overall survival (OS) rates. Suggesting that CTC profiling may serve as a predictive biomarker for immunotherapy benefit (Ref. 165). Clinical adoption does face challenges, however, with PD-L1 expression and other biomarkers on CTCs being highly heterogenous, and off-target toxicities caused by checkpoint inhibition (Ref. 166).

Monoclonal antibodies have become an important therapeutic option in the treatment of multiple cancers (Ref. 153). Intracellular adhesion molecule-1 (ICAM1) levels have been found to be higher in clusters of CTCs when compared to single CTCs. Although clusters of CTCs are rare, they are associated with poor clinical outcomes and offer 20 to 100 times greater metastatic potential than single CTCs. The overexpression of ICAM1 has been found to promote metastasis in the lungs and is correlated with poor survival in breast cancer patients. In orthotopic models, it was observed that anti-ICAM1 antibodies were able to inhibit the aggregation of CTCs, while reducing the spontaneous metastasis in the lungs, prompting further investigation (Ref. 167). However, ICAM1 targeting largely remains preclinical, with no major clinical study addressing this approach. Additionally, the heterogenous expression of ICAM1 across tumour subtypes (only present in 0.3% of HCCs) presents a significant challenge (Ref. 31).

### Nanotechnology-based approaches

Nanotechnology has become an essential biomedical science, presenting creative solutions for tumour treatment, specifically in the realm of targeted drug delivery systems (Ref. 168). The high tumour vasculature retention and permeability in the TME allows nanoparticles and macromolecules to have a relatively long half-life (Ref. 169). Through the manipulation of parameters like charge, molecular mass and particle size, nanocarriers allow therapeutic drugs to be delivered precisely to tumour areas with minimal damage to healthy tissues (Ref. 170). Breakthroughs in tumour-targeted systems, from overcoming physiological obstacles to bringing innovative diagnostic and therapeutic approaches to extend life expectancy, are made possible with the use of nanomaterials and techniques (Ref. 171).

Dual-targeting nanoparticles were designed to neutralize CTCs in the bloodstream and prevent metastasis (Ref. 172). Nanoparticles have displayed the ability to capture CTCs under shear stress from blood vessels and were effective in targeting and neutralizing CTCs in a lung metastasis mouse model (Ref. 173). EpCAM-targeting aptamer and tumour neovessel-targetable ligands were combined to create drug-loaded nanoplatforms for nanoparticle creation (Ref. 174). The anti-EpCAM ligands, Ep23 aptamer and K237 peptide, were loaded onto a single nanoplatform through the use of the drug Paclitaxel, allowing for the neutralization of CTCs while simultaneously damaging the primary tumour (Ref. 175). Dual targeting nanoparticles showed enhanced cellular uptake, elevated levels of apoptosis induction and higher levels of cell viability inhibition *in vitro*, demonstrating their effectiveness. From a translational perspective, dual-targeting NPs are a step towards precision therapy and allow for customization based on CTC profiles, allowing for patient-specific ligand combinations (Ref. 176). However, large-scale synthesis and reproducibility of multi-ligand NPs pose significant technical barriers, with off-target binding and systemic toxicity remaining concerns (Ref. 177).

Tumour-specific apoptosis-inducing ligand (TRAIL) was coupled with activated platelet membrane-coated silica nanoparticles. Silicon (Si) nanoparticles coated in platelet membranes express essential platelet surface glycans and proteins. This allows for efficient targeting of CTCs and avoidance of phagocytosis (Refs. 178, 179). When conjugated with TRAIL, these particles selectively induce apoptosis in CTCs and reduce metastatic lesions in murine models (Ref. 180).

Building on this concept, synthesized platelet-membrane-coated nanovesicles (PM-NVs) were loaded with Doxorubicin (DOX) and TRAIL. These nanovesicles effectively accumulated at the tumour site, enabling TRAIL to be delivered to cancer cells (Ref. 181). Additionally, they developed platelet membrane-coated glucan nanoparticles (NPs) loaded with bortezomib and tissue plasminogen activator (tPA), which targeted haematological malignancies. This was achieved by taking advantage of the unique affinity between P-selectin, which is present on the membrane surface of platelets and CD44, highly expressed on malignant cells and the bone marrow niche (Ref. 182). This nanomedicine prevented the formation of multiple myelomas, decreased off-target effects, increased the availability of the drug at the myeloma site, and simultaneously eliminated thrombotic repercussions in preclinical models (Ref. 180).

While these biomimetic platforms demonstrate strong potential for CTC treatment, challenges to translating preclinical results to clinical trials remain. The anti-cancer efficacy and safety have still not been fully established in humans. Additionally, the extrusion method for biomimetic NP synthesis is still hard to scale and quite time-consuming, while reproducibility still remains a challenge (Ref. 183).

### Small molecule therapeutics

Small-molecule inhibitors represent a major class of targeted anticancer therapeutics designed to modulate specific molecular dependencies that drive the initiation, progression and metastasis of tumours. Many cancers exhibit a reliance on dysregulated pathways governing proliferation, survival, metabolic adaptation, immune evasion and apoptosis (Ref. 184), which renders them susceptible to therapeutic treatments. Based on the nature of their molecular targets, selective small-molecule inhibitors can broadly be categorized into kinase and non-kinase inhibitors (Ref. 185).

Selective small-molecule kinase inhibitors have been explored for directly targeting CTC survival and metastatic ability. Recent work using lung cancer CTC cluster models demonstrated that CTC clusters exhibit increased resistance to epidermal growth factor receptor tyrosine kinase inhibitors (EGFR-TKIs) when compared to single or adherent tumour cells. Transcriptomic and functional analysis revealed that activation of the HGF/c-Met signalling axis is an important mediator for the survival of CTCs and their therapeutic resistance. The inhibition of c-Met using the small-molecule kinase inhibitor tivantinib suppressed the survival of lung cancer CTC clusters, while simultaneously reducing the CTC cluster-driven metastasis *in vivo* (Ref. 186).

Among non-kinase inhibitors, digoxin has emerged as a drug that targets metastasis-relevant mechanisms. Digoxin is a cardiac glycoside that inhibits the Na^+^/K^+^-ATPase, a membrane ion transporter that has been found to be involved in cell-cell adhesion and collective tumour cell behaviour. Preclinical studies demonstrated that inhibition of Na^+^/K^+^-ATPase disrupts CTC clusters by weakening their intracellular adhesion, leading to cluster disassociation and reduced metastatic seeding. These findings were translated into a first-in-human proof-of-concept study in patients with metastatic breast cancer, where digoxin treatment at 0.125-0.25 mg per day resulted in a significant reduction in mean CTC cluster size (Ref. 53).

### In vitro CTC-derived models for drug testing and mechanistic studies

An important aspect of CTC research is to study the mechanistic basis behind their properties and explore new CTC-based biomarkers and targeting strategies. The generation of *in vitro* models, such as CTC-derived cell lines, at relevant time periods of disease progression is crucial to both the functional and longitudinal characterization of these cells. Additionally, the demand for large-scale testing has promoted the development of tumour organoid-on-chips (Refs. 187, 188).

CTC *in vitro-*derived models have been established across multiple tumour types, such as breast, prostate, lung, colorectal and pancreatic cancer. These models primarily consist of short-term CTC cultures and CTC-derived cell lines, which are generated under defined culture conditions. While the fragility and rarity of CTCs make this technically challenging, successful *in vitro* expansion enables direct functional analysis of these cells. Additionally, CTC-derived cell lines often preserve key biological characteristics of the originating tumours, such as mutations and phenotypic plasticity (Ref. 189).

Beyond conventional CTC-derived cell lines, three-dimensional CTC-derived organoids have emerged as more advanced *in vitro* systems that accurately capture the structural, phenotypic and functional heterogeneity of metastasis. CTC organoids preserve key features such as epithelial-mesenchymal plasticity, stem-like states and therapy-resistant subpopulations that cannot be accurately portrayed in 2D cultures. These organoids also allow for functional analysis of metastatic ability, drug response and signalling responses in an environment more similar to its true biological state (Ref. 190). Building on these advances, microfluidic chip-based organoid cultures can stimulate the complex *in vivo* pathophysiological environment by providing precise control over the biochemical, physical and nutritional microenvironment. This enables more accurate drug testing and tumour modelling. Supported by microfluidic technology, CTC-derived organoids become highly dynamic and reproducible cancer models (Refs. 190, 191).

The use of CTCs in precision medicine has also been gaining momentum in recent years, with its use in adjuvant/ neoadjuvant therapies, the detection of recurrent diseases and its role as a possible pharmacodynamic biomarker. Advancements in this field could lead to the development of new personalized therapies.

## Challenges and future scope

### Challenges

CTCs have demonstrated great potential in the realm of cancer prognosis, diagnosis, monitoring and treatment. Identifying and examining these cells released from primary tumours provides valuable insights into the disease dynamics of cancer, thereby offering directions for tailored treatment plans. However, there are numerous challenges involved in detecting and isolating CTCs.

The role of CTCs may differ in different clinical conditions, due to which its roles in various cancers must be carefully studied. There is an increasing number of inconsistent results being reported in relation to the links between the observed levels in CTCs and the survival rate (Ref. 192). A lack of a standard protocol is also lacking with CTC-cantered prognostic or treatment modules, and currently, the only FDA protocol is the CellSearch© system (Ref. 193).

Another challenge regarding CTC detection is its scarcity in the bloodstream, as approximately only 1-100 CTCs, along with 10^6^-10^8^ RBCs, can be found per mL of blood (Ref. 194).

Biological questions, such as the nature of the factors determining the tendency of CTC to metastasize still haven’t been answered, along with which pathways could be targeted for inhibition to slow CTC metastasis. Regulation of CTCs by the circadian rhythm, due to which their distribution may not be uniform, is also a cause for concern (Ref. 74).

Many current studies focused on CTCs as a biomarker usually only investigate advanced or metastatic cancers, while rarely discussing early-stage cancers. It is important to understand if CTCs can reliably be used as a possible biomarker for early-stage cancers as well, as this can improve the prognosis of patients diagnosed with highly metastatic cancers, such as glioblastoma (Ref. 74).

### Future directions

Due to the scarcity of CTCs in early-stage cancers, maximizing the sensitivity of CTC detecting devices proves to be the most favourable option. Emerging approaches that utilize microfluidic devices, nanostructured capture surfaces and multiplex isolation strategies can aid in detection/isolation, provided that cell loss is accounted for. Correcting this issue or minimizing its effect could lead to the standardization of CTC detection technologies, allowing for more reproducible results and data comparability, which remains a major hurdle to clinical adoption (Ref. 195).

Enhancing the molecular characterization of CTCs may assist in their detection/isolation. Distinguishing between dormant and proliferative CTCs or single cells versus clusters could prove to provide major insights into CTC metastatic risk. This can be achieved through the identification of specific markers for CTC clusters or RNA sequencing of clusters of CTCs to identify the immune cells that interact with specific CTC clusters, and thereby quantify the effect they have on the cellular pathways, which are potential therapeutic targets (Ref. 196).

Future clinical utility will also depend on multi-analyte liquid biopsies, where CTCs and their associated biomarkers, such as circulating proteins, microRNAs and cell free tumour DNA (ctDNA), are analysed and targeted. Such assays have already shown preliminary success in increasing CTC sensitivity in both triple-negative breast cancer and prostate cancer (Refs. 195, 197).

These advances suggest that the future of CTC research does not rely on a single platform, but on integrated, standardized approaches that enable both sensitive detection and accurate biological interpretation.

## Conclusion

The increased diversity of tumour-associated cell populations complicates processes associated with disease diagnosis and treatment for various cancers. Key populations, such as CTCs and CSCs, remain areas of interest. CTCs are particularly important, owing to their association with poor patient prognosis, increased recurrence rates, increased likelihood of metastasis and drivers of immune evasion, amongst other functions. Techniques for CTC detection are hampered by their lack of wide applicability in clinical settings. However, advancements in this area, such as the decreased scale of operations and lowered cost due to the implementation of microfluidics and nanotechnology, have shown promising results. Isolation methods suffer from similar issues such as a lack of specificity and the sparse nature of CTCs, with size-based filtration and immunocapture being the primary technologies used in this area. Modifications that show improved clinical applicability, efficacy and scalability are purported to be essential for further improvements. Therapeutics targeting CTCs offer scope to improve overall survival, reduce chances of recurrence and metastasis, as well as improving patient quality of life post-treatment, as they can act as supplementary treatment modalities to existing chemotherapy modules, whose cytotoxicity can prove to be detrimental in cancers where the median age of diagnosis is higher. Further understanding of CTCs, related cellular mechanisms and effects on the tumour landscape could allow for their use in diagnosing early-stage cancers, enhanced molecular characterization of tumours. This could also aid in improving attempts at detection and isolation, while also improving treatment efficacy by utilizing them to identify and target other effective biomarkers in various cancers.
